# Newly Design Porous/Sponge Red Phosphorus@Graphene and Highly Conductive Ni_2_P Electrode for Asymmetric Solid State Supercapacitive Device With Excellent Performance

**DOI:** 10.1007/s40820-019-0360-3

**Published:** 2020-01-17

**Authors:** Nazish Parveen, Muhammad Hilal, Jeong In Han

**Affiliations:** 1 Department of Chemistry, College of Science, King Faisal University, Al-Ahsa, 31982 Saudi Arabia; 2 Flexible Display and Printed Electronic Lab, Department of Chemical and Biochemical Engineering, Dongguk University - Seoul, Seoul, 04620 Republic of Korea

**Keywords:** Sponge red phosphorus, Porous graphene, Ni_2_P, Electrode, Asymmetric solid-state supercapacitor

## Abstract

Supercapacitors have attracted much attention in the field of electrochemical energy storage. However, material preparation, stability, performance as well as power density limit their applications in many fields. Herein, a sponge-like red phosphorus@graphene (rP@rGO) negative electrode and a Ni_2_P positive electrode were prepared using a simple one-step method. Both electrodes showed excellent performances (294 F g^−1^ and 1526.6 F g^−1^ for rP@rGO and Ni_2_P, respectively), which seem to be the highest among all rP@rGO- and Ni_2_P-based electrodes reported so far. The asymmetric solid-state supercapacitor was assembled by sandwiching a gel electrolyte-soaked cellulose paper between rP@rGO and Ni_2_P as the negative and positive electrodes. Compared to other asymmetric devices, the device, which attained a high operating window of up to 1.6 V, showed high energy and power density values of 41.66 and 1200 W kg^−1^, respectively. It also has an excellent cyclic stability up to 88% after various consecutive charge/discharge tests. Additionally, the device could power commercial light emitting diodes and fans for 30 s. So, the ease of the synthesis method and excellent performance of the prepared electrode materials mat have significant potential for energy storage applications.

**Electronic supplementary material:**

The online version of this article (10.1007/s40820-019-0360-3) contains supplementary material, which is available to authorized users.

## Highlights

- Three-dimensional phosphorus@graphene is established as a negative electrode and Ni_2_P as a positive electrode for asymmetric supercapacitor.
- The supercapacitor shows higher performance than those of all Ni_2_P-based electrodes, which has an extended operating voltage window of 1.6 V, energy density of 41.66 Wh Kg^−1^ and rate capability of up to 88% after 5000 cycles.

**Electronic supplementary material:**

The online version of this article (10.1007/s40820-019-0360-3) contains supplementary material, which is available to authorized users.

## Graphical abstract

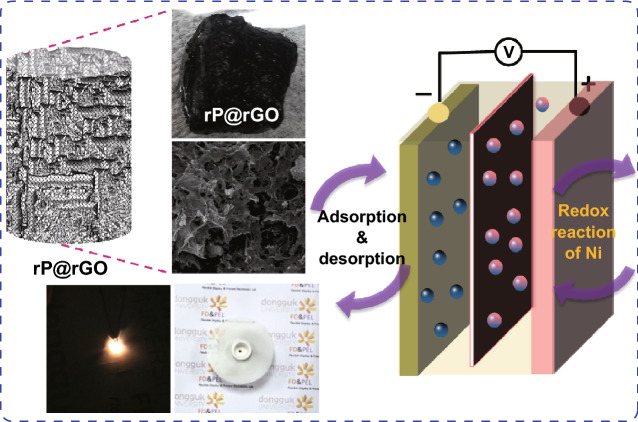

## Introduction

In recent years, the use of portable electronic devices, including smart phones and laptops, has been considered a desirable approach to the advancement of human life [1, 2]. However, an increasing world population and an increasing demand for essential needs may lead to a future energy crisis [1–4]. To overcome such a challenge, many studies have been conducted to develop novel energy storage devices, including lithium ion batteries, sodium ion batteries, and supercapacitors [1–5]. Owing to their higher power densities, fast charge/discharge rates, and longer life span, supercapacitors, which bridge the gap between physical capacitors and batteries, are considered to be promising energy storage devices [6–8]. To obtain supercapacitors with enhanced performance, researchers are devoted to developing/engineering metal oxide, sulfide, and hydroxide nanoparticles, as well as carbonaceous materials that are more suited to and efficient for use in supercapacitors [5, 7–9]. Even though numerous studies have been performed to develop high energy and power density supercapacitors controlled by a capacitive electrode, a number of challenges still exist [2–4]. The hybrid supercapacitor electrode-based concept, which combines a battery-type and a capacitor-type electrode to achieve high energy and power densities, has been proposed [6–8]. However, mostly *p*-type semiconductor pseudo-capacitors, which are kinetically unfavorable to the fast electron transport required for high power density delivery, have been used [7–10]. Consequently, transition metal compounds have received a lot of attention recently, owing to their metalloid properties and excellent electrical conductivities [2, 4–9]. Among them, nickel phosphide (Ni_2_P) has attracted the most attention because of its superior electrical conductivity and intrinsic metallic nature, which permit fast electron transfer at the electrode/electrolyte interface [2, 7–13]. Additionally, it has a high theoretical capacitance value of 2601 F g^−1^ [12, 15]. However, few studies have reported its use as an electrode in half-cell and full-cell assemblies that show better electrochemical supercapacitance performance and energy density [12, 14–20].

To obtain high energy and power densities, the development of negative carbonaceous electrodes is also important [19, 21]. Various types of materials have been targeted for developing negative electrodes for use in hybrid supercapacitor devices; e.g., owing to their high power density, large surface area, and long life cycle, carbon- (CNT, AC) and graphene-based materials have been extensively employed as the most preferred electrode materials for electric double-layer capacitors (EDLC) [18, 19, 22]. Furthermore, graphene and CNT are considered to be good candidates owing to their large surface area and chemical stability [19, 22]. To achieve a higher electrochemical supercapacitive performance, various other composite electrode materials have been developed by combining graphene with V_2_O_5_, Fe_2_O_3_, and MoO_3_ [23, 24]. However, the specific capacitance and power density values obtained were unsatisfactory, making further investigation to develop a new negative electrode material necessary [23]. In this light, considering its abundance in the earth’s crust and its high theoretical specific capacity, great progress has been made in the development of red phosphorus (rP)-based anode material for use in batteries [25, 27]. However, like its composite materials, it has not yet been used as the negative electrode in asymmetric supercapacitors. Hence, its application in this context might open a new window for the development of advanced negative electrodes in the field of energy storage devices [21, 26]. Apart from the challenges associated with the positive and negative electrodes, the use of liquid or electrolyte-based asymmetric supercapacitive devices is still an issue that affects the overall performance of the supercapacitor. Compared to liquid or electrolyte-based asymmetric supercapacitors, their solid-state counterparts have many advantages, including portability, flexibility, and environmental friendliness [12, 22].

The objective of this study was to investigate and develop a solid-state asymmetric device containing specific positive and negative electrodes. For attaining this objective, a porous sponge-like red phosphorus@graphene electrode (rP@rGO), which was used for the first time as the negative electrode in a solid-state asymmetric supercapacitive device, was developed. Based on the existing literature, this study is the first to report the use of this material as a negative electrode in a solid-state asymmetric supercapacitive device. The basic spectroscopic and microscopic characteristics of this electrode, as well as its individual electrochemical performance, were studied in detail, before the asymmetric solid-state device was assembled. At 1.5 A g^−1^, this negative electrode showed a capacitance of 294 F g^−1^, which is approximately higher than those of other electrodes that have reported in the previous studies [19, 21]. Additionally, without using any surfactant, a positive electrode (Ni_2_P) was developed using simple steps, considering the abundance of its precursor, red phosphorus (rP). The performance of the Ni_2_P was tested in a three-electrode assembly cell, and at 1 A g^−1^, it was found to show a high capacitance of 1526.66 F g^−1^, which seems to be the highest capacitance value reported in the previous studies on the use Ni_2_P as a positive electrode (Table S1). After the study of the individual basic electrochemical properties of this positive electrode in a three-electrode assembly, the solid-state asymmetric supercapacitive device was assembled by sandwiching a gel electrolyte-soaked cellulose paper with the positive and negative electrodes. The asymmetric device, which resembled commercial devices extended the potential voltage window up to 1.6 V, showed a maximum energy density of 41.66 Wh kg^−1^ at a power density of 1200 W kg^−1^, as well as a good rate capability and columbic efficiency of up to 5000 cycles. The assembled hybrid device was also capable of powering commercially available light emitting diodes (LEDs) and fans for 30 s, highlighting the possibilities of its potential application.

## Experimental Procedure

### Materials

Red phosphorus was purchased from Yakuri Pure Chemicals (Kyoto, Japan), whereas nickel nitrate hexahydrate and polyvinylidene fluoride (PVDF) were obtained from Sigma-Aldrich (South Korea). The aqueous medium used in these experiments was de-ionized water obtained from 30 PURE ROUP purification system.

The morphology and structure of rP@rGO and Ni_2_P were observed using a HITACHI-S4800 scanning electron microscope (SEM) and a Tecnai G2 F20 field emission transmission electron microscope (FE-TEM, FEI, USA). Their crystal structures were analyzed using X-ray diffraction (XRD, PANalytical, X’Pert-PRO MPD, Netherlands) with Cu K*α* radiation (*λ* = 0.15405 nm). Their oxidation sates and surface compositions were determined using X-ray photoelectron spectroscopy (XPS, ESCALAB 250 XPS system, Thermo Fisher Scientific, UK). Additionally, the determination of their electrochemical properties through cyclic voltammetry (CV) and charge/discharge (CD) measurements was performed using a potentiostat (Versa STAT 3, Princeton Research, USA).

### rP@rGO Synthesis

The porous sponge-like rP@rGO was prepared using a modified Hummer’s method. A graphene solution (1 mg mL^−1^) was sonicated for 10 min. Thereafter, rP (70 mg) was added, and the mixture was further sonicated for 60 min. The mixture was then transferred into a Teflon-lined autoclave, properly sealed, and kept for 12 h at 180 °C. After the reaction was completed, the product obtained was allowed to cool down to room temperature naturally, and finally a sponge-like rP@rGO framework was obtained. It was then washed with water and ethanol, and dried in a vacuumed oven for 12 h (Fig. S1).

The treated rP particles were dispersed in the graphene solution inside the Teflon-lined autoclave, until the temperature was increased to 200 °C. This temperature increase led to the generation of bubbles owing to the boiling water, and the rP particles gradually assembled around these bubbles. The gradual increase in the size of the bubbles made it possible for more rP particles to be distributed all over the gas bubbles, which finally separated from the rP, forming pores inside the rP [1].

### Ni_2_P Synthesis

To synthesize Ni_2_P, rP was first treated in a Teflon-lined autoclave at 200 °C to remove the oxide layer on its surface. The treated rP was then dispersed in water using ultra-sonication, after which different ratios of the nickel precursor were added. The mixture was then sonicated for 10 min, after which it was transferred into a Teflon-lined autoclave and heated at 200 °C for 12 h. After the reaction was complete, the product was allowed to cool down to room temperature naturally, and the black precipitate obtained was further extracted and washed several times with distilled water. The samples obtained were then dried at 80 °C using vacuumed oven. (The different ratios of rP and the nickel precursor used to prepare the samples were 1:0.5 and 1:1 for Ni_2_P-1 and Ni_2_P-2, respectively.)

### Electrochemical Measurements in a Three-Electrode Assembled Cell

The electrochemical properties of the positive Ni_2_P and the negative rP@rGO electrodes were measured using different methods, including cyclic voltammetry (CV), charge discharge (CD), and electrochemical impedance spectroscopy (EIS) measurements in a three-electrode system, using a freshly prepared 2 M KOH aqueous solution as electrolyte. The prepared samples, saturated AgCl/Ag, and a platinum plate were used as the working, reference, and counter electrodes, respectively. Working electrodes were obtained by preparing a slurry of the active materials (i.e., 80% rP@rGO or Ni_2_P, 10% carbon black, and 10% PVDF) in N-methyl-2-pyrrolidone (NMP) as solvent. The resulting slurry was then used to coat commercially available nickel foam, in the case of Ni_2_P and carbon paper in case of rP@GO (coating area = 1 cm^2^), after which the prepared electrodes were dried in oven at 80 °C for 12 h. All the electrochemical tests were then performed at room temperature.

### Assembling the Solid-State Asymmetrical Supercapacitor

Slurries of the two working electrodes were prepared by mixing the activate materials (i.e., 80% rP@rGO or Ni_2_P, 10% PVDF, and 10% carbon black) using NMP as solvent. The resulting mixtures were then powdered using a mortar and pestle for 10 min. The obtained slurries were then used to coat commercially available carbon paper and nickel foam, for rP@rGO and Ni_2_P, respectively (effective coating area = 1 × 3 cm^2^). The coated electrodes were further dried in oven for 12 h, after which the solid-state symmetrical supercapacitor was assembled by sandwiching a gel electrolyte-soaked cellulose filter paper between the electrodes coated with the active material, i.e., Ni_2_P and rP@rGO for the positive and negative electrodes, respectively (Fig. S2). The gel electrolyte (PVA-KOH) was prepared by dissolving 10 g of polyvinyl alcohol in a 1 M KOH aqueous solution.

## Results and Discussion

**Fig. 1:**
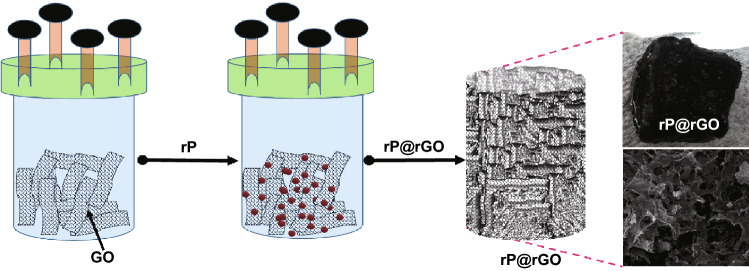
Steps involved in the synthesis of the rP@rGO sponge

Figure 1 shows the steps employed in the synthesis of the sponge-like rP@rGO framework. The GO was diluted in an aqueous medium, treated rP was added, and the mixture was subjected to further ultra-sonication. During the hydrothermal treatment, GO was converted to rGO sheets, which assembled into sponge-like morphology. Simultaneously, rP was also distributed on the surface of the assembled rGO sheets. At the end of the reaction, a sponge-like 3D porous rP@rGO frame was obtained without using the freeze-dry method.

**Fig. 2:**
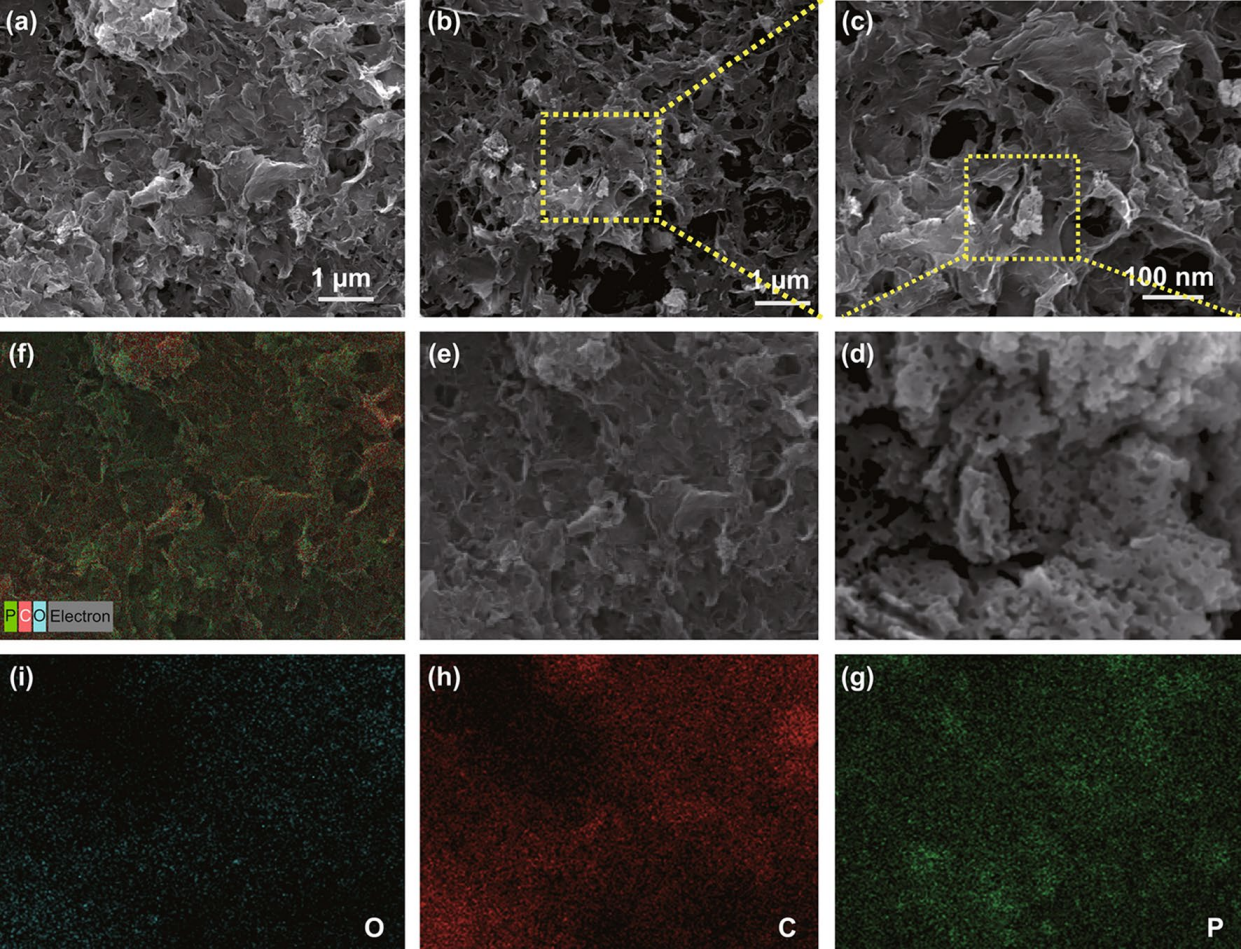
**a**–**d** SEM images at different magnifications. **e–i** Corresponding rP@rGO mapping images

These mechanisms were further supported by SEM and TEM analyses (Figs. 2 and S3). The surface morphology and structure of the rGO and rP@rGO nanocomposites were observed using SEM (Figs. 2a–d and S3a, b) and corresponding mapping (Fig. 2e–i), respectively. rGO showed a porous wrinkled sheet surface, and the presence of the rP sheet made it more porous. Additionally, the irregular size of the rP composites with visible holes trapped in the rGO could be clearly observed, as shown in Fig. 2d, and the corresponding elemental mapping profiles clearly showed that the rP particles were well-distributed over the rGO. These trapped rP particles could improve electrical contact between the individual constituents.

**Fig. 3:**
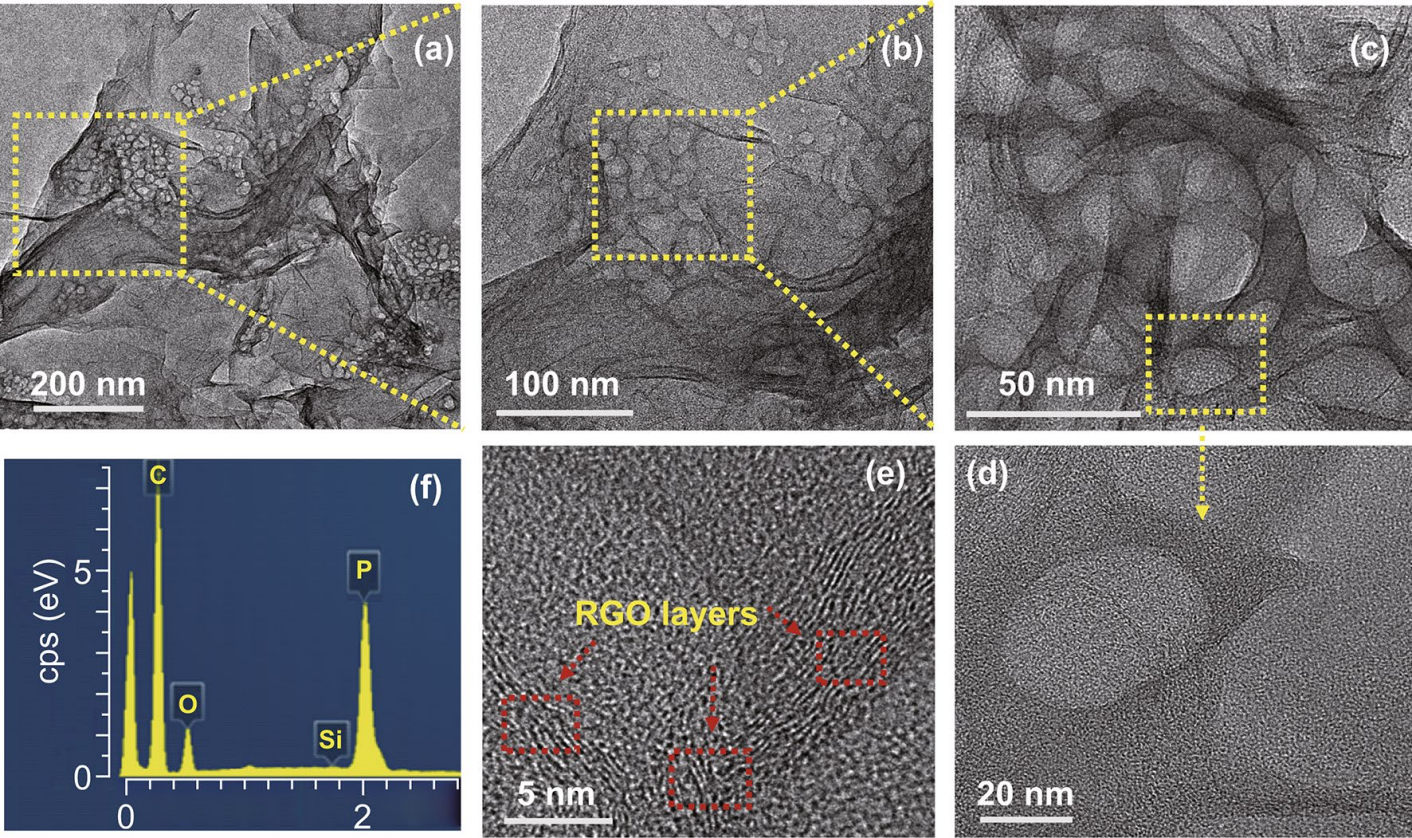
**a**–**e** TEM and HRTEM images at different magnification. **f** Corresponding rP@rGO EDX graphs

The porous structures and holes in rP were further confirmed by TEM (Fig. 3a–c) and HRTEM (Fig. 3d–e) images. Pores with sizes ~ 8–10 nm were present in the rP@rGO nanocomposite. HRTEM images also showed that rP composites with holes were incorporated into the rGO sheets, and showed a strong interpenetrated interface. Furthermore, detailed HRTEM images of the rP@rGO electrode showed an 8–10-layer rGO thickness (Fig. 3e). The corresponding EDX analysis clearly showed the absence of any impurities, and confirmed the presence of carbon, oxygen, and phosphorous (Fig. 3f). Generally, such morphology and porous behavior evidently make the electrode surface accessible to the electrolyte, thus facilitating rapid charge transportation during the electrochemical process.

**Fig. 4:**
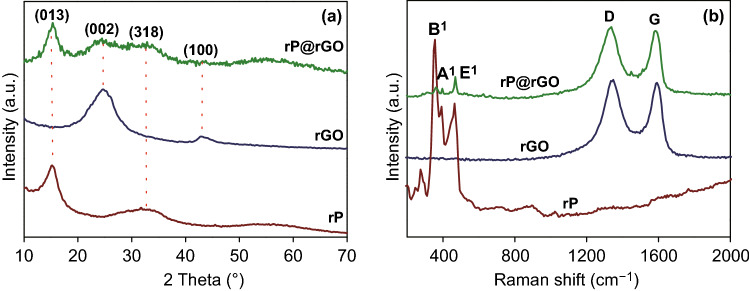
**a** XRD pattern and **b** Raman spectra of rP, rGO, and rP@rGO

The structural properties of the B-rGO, rP, and rP@rGO nanocomposites were easily examined using XRD (Fig. 4a). The well-matched rP diffraction pattern was observed at 2*θ* ~ 15°, while a broad peak was observed at ~ 32.8°. These diffraction peaks were readily attributed to the monoclinic phase of rP, which is well-matched with JCPDF card No. 44-0906. Conversely, a broad peak observed at 2*θ* ~ 25° was attributed to the (002) plane (JCPDF card No. 75-1621), clearly indicating the formation of rGO from GO during the hydrothermal treatment. These peaks were also observed with rP@rGO nanocomposites, clearly indicating the successful formation of the nanocomposite [1, 27]. Furthermore, the diffraction patterns of rP in the rP/rGO nanocomposite at 2*θ* ~ 15° were still visible, confirming that hydrothermal treatment did not destroy the crystalline behavior of rP; this might be an advantage of its electrochemical performance.

In accordance with previously reported data [13, 27], the characteristic Raman peak of rP, observed between 200 and 500 cm^−1^ (Fig. 4b), was attributed to B1, A1, and E1 modes. The two characteristic peaks observed at ~ 1350 and ~ 1599 cm^−1^ were attributed to the D and G bands of the well-prepared rGO [27]. After the analysis of the rP/rGO nanocomposite, these characteristic rP and rGO peaks were still clearly visible. However, the rP peak intensity was lower, clearly indicating the successful formation and confinement of rP in rGO sheets.

**Fig. 5:**
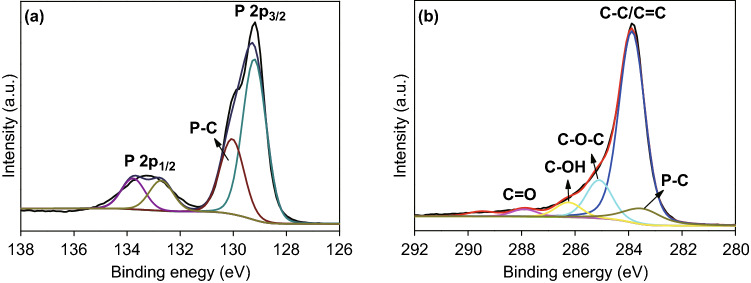
**a** P 2*p* and **b** C 1*s* high-resolution rP@rGO XPS spectra

XPS analyses were performed to determine the chemical composition, surface characterization, and percentage of rP and rGO in the prepared rP@rGO samples. The XPS spectra (Fig. S4) provided a broad view of the surface elemental composition of the rP@rGO composites. Figure 5 shows the chemical behavior and chemical bonding of the rP@rGO nanocomposites. The major peaks observed at a binding energy (BE) of 129.29 ± 0.02 and 133.2 ± 0.02 correspond to the 2 P_3/2_ and 2 P_1/2_ doublet, while the peak observed at a BE of 130.08 ± 0.02 was attributed to the presence of P–C bonds (Fig. 5a). This binding of phosphorus to graphene carbon atoms prevented the conductive behavior of the electrode during the electrochemical reaction [13]. Additionally, the presence of P–C observed at a BE of ~ 283.58 eV bonds was further confirmed by the C 1*s* XPS spectrum (Fig. 5b) [27].

Apart from P–C bonds, C–O and C=O bonds as well as the non-oxygenated C–C bond were also observed during the analysis of the nanocomposite. Figure S5 shows the O 1*s* spectrum of the nanocomposite, which was deconvoluted into two peaks, located at BE of ~ 531.5 and ~ 533.5 eV, corresponding to the presence of hydroxyl and P–C–P bonding, respectively. The presence of these bonds is due to the interfacial interaction between the phosphorus and oxygen atoms in the graphene nanocomposite [27].

**Fig. 6:**
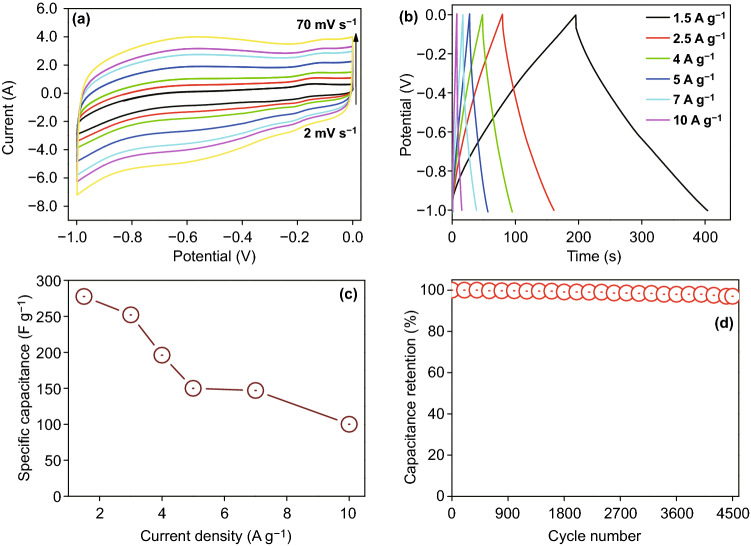
**a** CV, **b** GCD, **c** specific capacitance at different current loads, and **d** cyclic stability of the rP@rGO electrode

The initial electrochemical supercapacitive behavior of the negative electrode, i.e., rP@rGO was investigated using cyclic voltammetry (CV) within a scan range of 2–70 mV s^−1^, and charge/discharge measurements (CD) within a current load range of 1.5–10 A g^−1^, and the results are given in Fig. 6a. The rP@rGO CV curve showed exactly the normal behavior of an electric double-layer capacitor, and even at a higher scanning rate, the curve shape remained the same, indicating that the rP@rGO electrode has an excellent stability. This electric double-layer capacitance behavior was also supported by the CD profile (Fig. 6b), which showed that all the CD curves were almost symmetrical and triangular in shape. Furthermore, this behavior facilitates the enhancement of charge/discharge reversibility during electrochemical reactions.

The specific capacitance of the rP@rGO electrode at different current loads was also calculated and graphically shown in Fig. 6c, which shows that the highest capacitance (294 F g^−1^) was observed at 1.5 A g^−1^, and at 2.5, 4, 5, 7, and 10 A g^−1^, capacitance values of 225, 200, 150, 147, and 120 F g^−1^ were observed, respectively. Additionally, the calculated specific capacitance of the rP@rGO electrode was higher than those reported in the previous studies [19, 21, 28]. Apart from CV and CD measurements, the cyclic stability of the rP@rGO electrode was also measured during consecutive 4500 charge/discharge cycles at a current density of 5 A g^−1^. The results showed that it retained 97% of its initial capacitance, further highlighting its cyclic stability (Fig. 6d).

Ni_2_P synthesis, which employed simple steps and used a single procedure, can be explained as Eqs. 1–4: nickel nitrate does not react directly with phosphorus. Thus, rP is first reacted with water to form hypophosphorous acid that reduces the nickel ions in the nitrate to nickel atoms. The nickel atoms then react with the unreacted rP, forming the Ni_2_P [29].

1 $$ \left[ {{\text{Ni}}\left( {{\text{H}}_{2} {\text{O}}} \right)_{6} } \right]\left( {{\text{NO}}_{3} } \right)_{2} \to \left[ {{\text{Ni}}\left( {{\text{H}}_{2} {\text{O}}} \right)_{6} } \right]^{2 + } $$

2 $$ \left[ {{\text{Ni}}\left( {{\text{H}}_{2} {\text{O}}} \right)_{6} } \right]^{2 + } + {\text{H}}_{2} {\text{PO}}_{2}^{ - } \to {\text{NiOH}}^{ + } $$

3 $$ {\text{NiOH}}^{ + } + {\text{H}}_{2} {\text{PO}}_{2}^{ - } \to {\text{Ni}} $$

4 $$ 2 {\text{Ni}} + {\text{rP}} \to {\text{Ni}}_{2} {\text{P}} $$

**Fig. 7:**
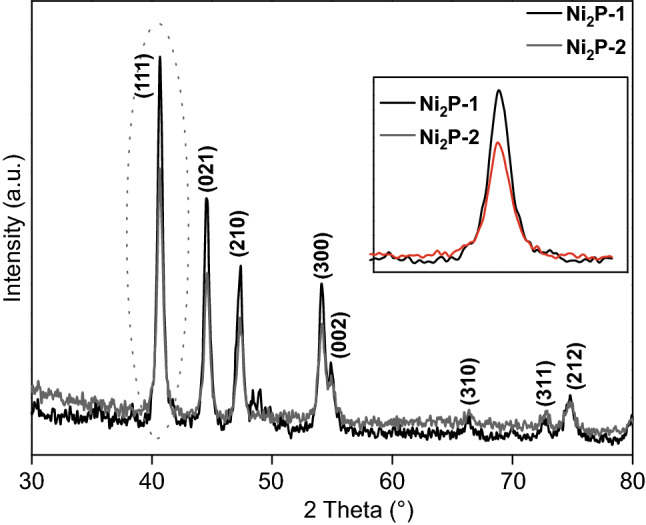
Ni_2_P-1 and Ni_2_P-2 XRD patterns

The crystal structure of Ni_2_P samples with different nickel precursor concentrations was examined using XRD, and the results are given in Fig. 7. The diffraction patterns of Ni_2_P-1 and Ni_2_P-2 showed peaks at 40.8°, 44.6°, 47.3°, and 54.2° which can be attributed to the (111), (201), (210), and (300) planes of the hexagonal phase of the Ni_2_P (JCPDF card No. 03-0953), respectively [16, 18]. No other peaks resulting from other phases were observed, confirming the purity of the samples. The diffraction peaks of the Ni_2_P samples were also very sharp and strong, showing that their crystallinity was not destroyed during the hydrothermal treatment. However, after increasing the nickel precursor concentration (Ni_2_P-2), the crystallite size decreased as shown in the inset of Fig. 7.

**Fig. 8:**
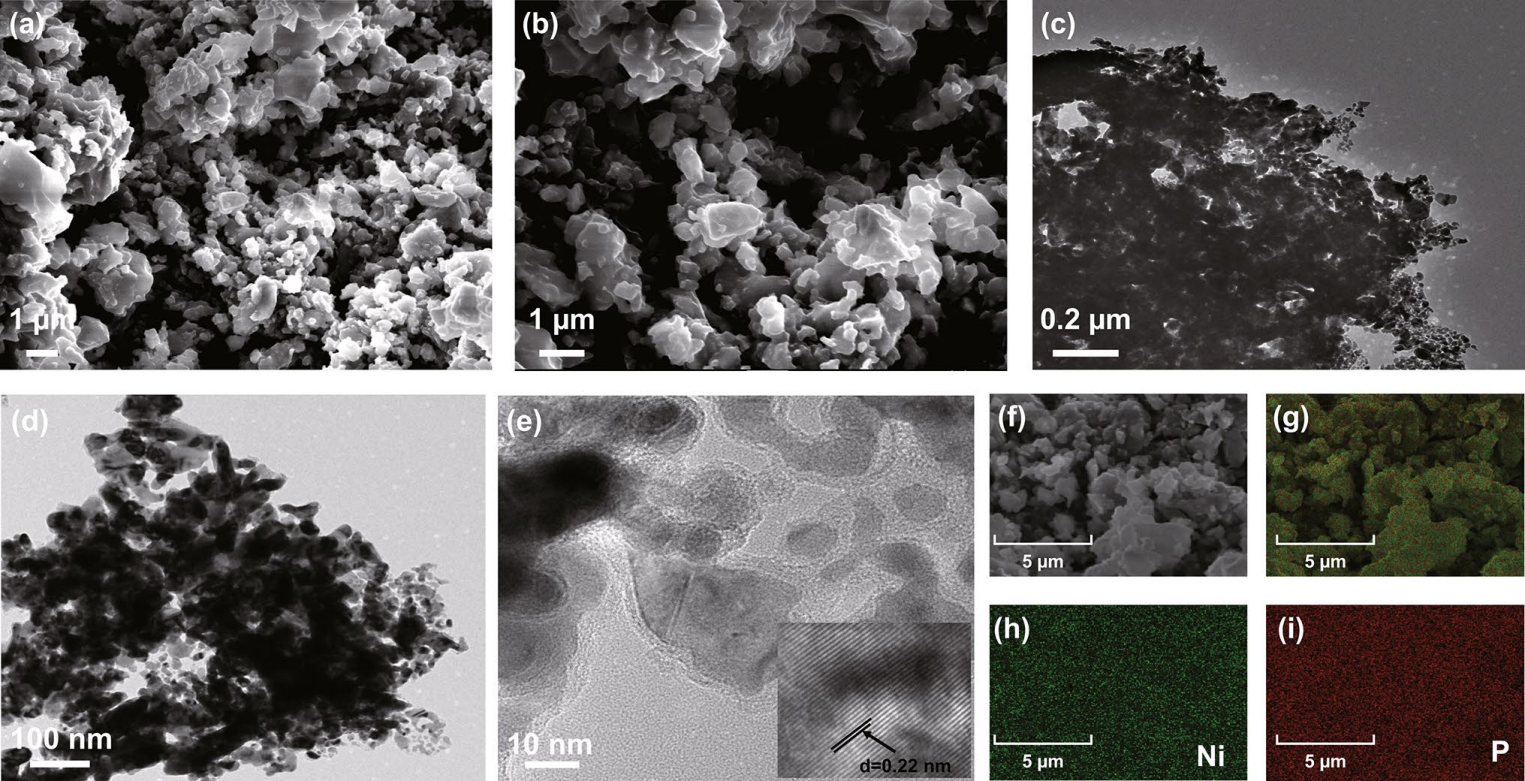
**a**, **b** SEM images, **c–e** TEM and HRTEM images, and **f** Corresponding Ni_2_P-2 elemental mapping

Ni_2_P morphology and structure were investigated using FESEM, TEM, and HRTEM, and the results are shown in Figs. 8 and S6. Ni_2_P-1 and Ni_2_P-2 SEM images at different magnifications, shown in Figs. 8a, b and S6a, b, revealed that Ni_2_P is composed of irregular and agglomerated nanoparticles. Additionally, after increasing the nickel precursor content, the size of the Ni_2_P-2 nanoparticles slightly reduced, an observation that was consistent with the XRD analysis. This aggregation of Ni_2_P particles was expected given their smaller size and higher surface activity. Ni_2_P TEM analysis showed that it consisted of a large number of aggregated nanoparticles with average size ranging between 20 and 30 nm (Fig. 8c, d), whereas the HRTEM results revealed that the clear lattice fringes with 0.22 nm spacings corresponded to the (111) plane of the hexagonal Ni_2_P structure (Fig. 8e). The corresponding Ni_2_P-1 (Fig. S6d–f) and Ni_2_P-2 (Fig. 8f–i) elemental mappings also showed a uniform distribution of nickel and phosphorus atoms in Ni_2_P.

The electronic state of the Ni and P in Ni_2_P-2 was determined from the XPS results shown in Fig. S7. The two main peaks observed at BEs of ~ 852.5 and ~ 870 eV corresponded to the spin orbit of the Ni_2_P doublet (Ni 2*p*_3/2_ and Ni 2*p*_1/2_). These peaks (Fig. S7a) were attributed to the presence of metallic Ni in Ni_2_P, whereas the peaks observed at BEs of ~ 856.2 and ~ 874.10 eV were attributed to the oxidized forms of Ni in the samples, a finding that is obviously consistent with the previous reports [12, 20]. Figure S7b represents the high-resolution P 2*p* XPS spectrum. It shows two peaks at BEs of ~ 129.3 and ~ 133.4 eV, which correspond to P 2*p*_3/2_ and P 2*p*_1/2_, respectively, attributed to Ni–P bonds and to the presence of phosphates on the Ni_2_P surface.

**Fig. 9:**
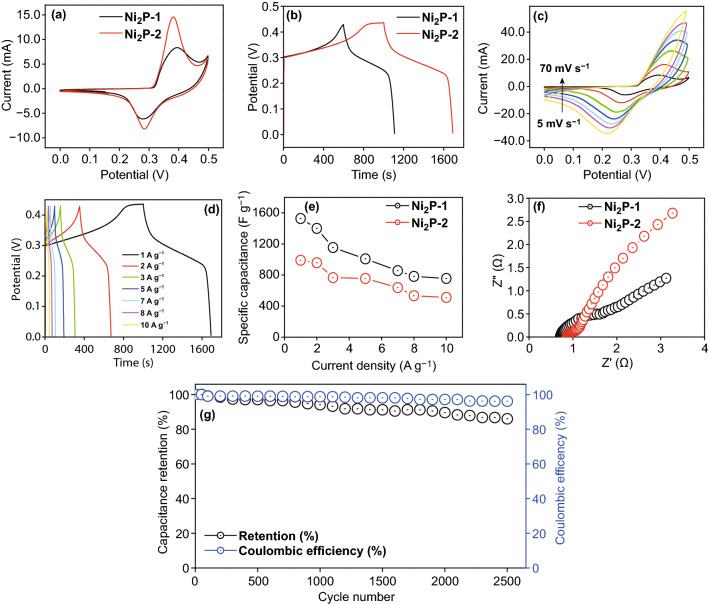
a Comparative Ni_2_P-1 and Ni_2_P-2 CV curves at a fixed scan rate. **b** Comparative Ni_2_P-1 and Ni_2_P-2 GCD curves at a fixed current load. **c** Ni_2_P-2 CV curves at different scan rates. **d** Ni_2_P-2 GCD curves at different current loads. **e** Calculated Ni_2_P-1 and Ni_2_P-2 specific capacitance. **f** Ni_2_P-1 and Ni_2_P-2 EIS spectra. **g** Capacitance and coulombic efficiency retention of Ni_2_P-2

The behavior of the Ni_2_P positive electrode was observed using CV and galvanostatic charge discharge (GCD) measurements (Figs. 9a–g and S8a, b), which are usually applied to establish the initial capacitive behavior of electrode materials [30, 31]. The Ni_2_P CV curve was recorded over a scan range of 5–70 mV s^−1^, and a potential range of 0–0.5 V (Figs. 9a, c and S8a). The comparative CV profile of the positive electrode clearly showed a redox peak, indicating that a strong faradic redox reaction occurred on the electrode surface, due to the reversible reaction involving Ni^2+^/Ni^3+^ interconversion, occurring inside the electrochemical cell on the electrode/electrolyte interface (Fig. 9a), i.e., as shown in Eqs. 5 and 6, the Ni in Ni_2_P can easily be oxidized to formed Ni(OH)_2_ in the presence of KOH [32–34].

5 $$ {\text{Ni}}^{2 + } + 2 {\text{OH}}^{ - } \to {\text{Ni}}\left( {\text{OH}} \right)_{2} $$

6 $$ {\text{Ni}}\left( {\text{OH}} \right)_{2} + {\text{OH}}^{ - } \to {\text{NiOOH }} + {\text{H}}_{2} {\text{O }} + {\text{ e}} $$

From the above reaction, it can be concluded that the charge and discharge forms of Ni were NiOOH and Ni(OH)_2_, respectively. Additionally, it was observed that the anodic peak shifted toward the positive potential, while the cathodic peak shifted toward the negative potential with the different scan rates. Peak current also increased linearly, suggesting a fast rate for the interfacial faradic reaction, and electronic and ionic transportations, at different scan rates. Figure 9a reveals that Ni_2_P-2 showed a stronger redox peak and larger integrated area under in the current potential curve compared to Ni_2_P-1, indicating a faster electrochemical reaction, owing to the higher Ni concentration in Ni_2_P.

After the initial CV measurement, the most appropriate GCD technique was employed to characterize and calculate the specific capacitance of the positive electrode material at different current densities, and the results obtained are depicted in Figs. 9b, d, and S8b. The comparative GCD (Fig. 9b) profile showed a visible plateau region, clearly indicating the faradic behavior of the electrode, an observation that is also consistent with the results of the CV measurements (Fig. 9c), as well as other previous reports on Ni_2_P-based electrode. The resulting specific capacitance of the positive electrode with respect to the GCD profile was 1526.66, 1400, 1053.33, 977.77, 855.55, 782.22, and 755.55 F g^−1^ at current loads of 1, 2, 3, 5, 7, 7, 8, and 10 A g^−1^, respectively, for Ni_2_P-2, and 980, 977.77, 766.66, 755.55, 637.77, 533.33, and 511.11 F g^−1^ at current loads of 1, 2, 3, 5, 7, 7, 8, and 10 A g^−1^, respectively, for Ni_2_P-1 (Figs. 9d and S8b).

From Fig. 9e, it is obvious that the maximum specific capacitance of the electrode is 1526.66 F g^−1^ at current density of 1 A g^−1^, which is almost higher than the specific capacitance of the Ni_2_P electrode reported in the previous studies (Table S1). These higher capacitance values were obtained even at a current load as high as 10 A g^−1^ suggesting that the stability of the electrode is significant. The electron transfer and interfacial properties of the Ni_2_P electrode were investigated using EIS measurements (Fig. 9f). A straight line with small slope, which generally represents the electrolyte diffusion and capacitance nature of the electrode, was observed in the low-frequency region, whereas a semicircular arc, which generally represents the contact resistance at the electrolyte/electrodes interface, was observed in the high-frequency region. Figure 9f, which presents the EIS plots for Ni_2_P-1 and Ni_2_P-2, shows a semicircular arc for Ni_2_P-2 that was smaller than that of Ni_2_P-1, indicating the lower charge transfer resistance on the Ni_2_P electrode. This observation might be attributed to the high electron transport property and conductive behavior of Ni_2_P-2 compared to Ni_2_P-1. These results were also consistent with CV and CD results.

The stability of the positive electrode was measured using several consecutive charge/discharge cycles of the representative voltage window at a current load of 10 A g^−1^. Figure 9g shows that the capacitance value did not drop quickly, given that after 2500 cycles, it was still at 86.3% of initial value. The representative capacitive performance of the Ni_2_P electrode was compared to those reported in the previous studies, and it was found to exhibit the highest capacitance (Table S1).

After the detailed and successful sequential measurement of the electrochemical properties of the positive and negative electrodes in a common three-electrode assembly cell, the asymmetric supercapacitor device was assembled by sandwiching an electrolyte gel-soaked cellulose filter paper with both electrodes, as shown in Fig. S2. To obtain an optimal performance for both electrodes, we controlled their mass loadings by adjusting the mass of the active material [2]. From an application point of view, the CV and GCD measurements of the assembled device were investigated using a wide range of operational potential windows (0–1.6 V). The potential window of the positive electrode only ranged between 0 and 0.5 V (Fig. 9b), whereas that of the negative electrode ranged between 0 and − 1 V (Fig. 6b). However, after combining these two electrodes, the potential window significantly increased up to 1.6 V, highlighting the advantage of the assembled device [35–38].

**Fig. 10:**
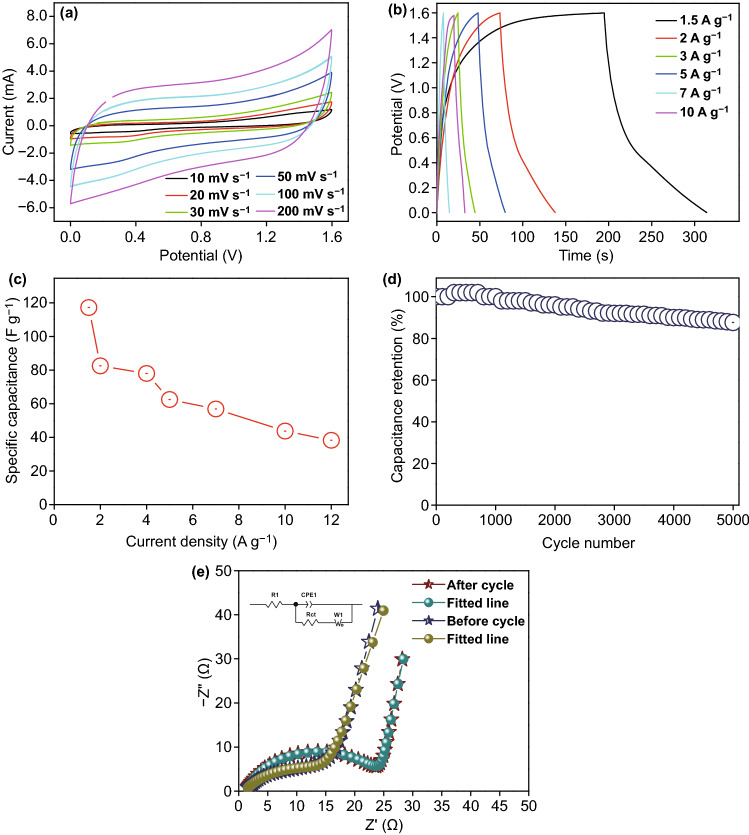
**a** CV profile of the assembled device at various scan rates. **b** Galvanostatic charge/discharge profile of the device at various current densities. **c** Specific capacitance of the assembled device at various current loads. **d** Cycling stability test of the device in the inset showing the GCD profile of the last complete cycle. **e** Nyquist plot of the assembled device before and after the cycling stability test (inset: circuit)

The electrochemical performances of the assembled asymmetric supercapacitor device at different scan rates and current loads were further evaluated using CV and CD, respectively. The CV profile (Fig. 10a) of the assembled device clearly showed that it exhibited the typical behavior of a hybrid asymmetric supercapacitor, meaning that both pseudo-capacitive and electric double-layer capacitive characteristics were exhibited by the device. Additionally, the shape of its CV profile practically remained constant during the high scan measurements, which is also an advantage of the assembled electrodes. In Fig. 10b, all the CD curves of the device showed an obvious discharge plateau, indicating that a redox reaction had occurred at the surface of the electrode in the presence of KOH-based gel electrolyte, owing to the pseudo-capacitive behavior of the device. Its specific capacitance calculated from the GCD curve and using the equation of the assembled asymmetric supercapacitor device were 117.18, 82.5, 78, 62.5, 56.87, 43.75, and 38.25 F g^−1^ at current densities of 1.5, 2, 3, 5, 7, 10, and 12 A g^−1^, respectively (Fig. 10c). The capacitive performance of the devices was higher than those reported in the previous studies on Ni_2_P- and rGO-based assembled asymmetric devices (Table 1).

**Table 1:** Performance comparison of the Ni_2_P//rP@rGO asymmetric supercapacitor device with data from the previously published studies

Sample no.	Asymmetric assembled devices	*C*_sp_ (F g^−1^)	Energy density (Wh kg^−1^)	Power density (W kg^−1^)	Retention (%)	Cycle no.	References
1	AC//Ni–P	105	29.2	400	84.5	1000	[10]
2	AC/NaNiPO_4_	56	20	138	70	500	[5]
3	Fe_2_O_3_//Ni_2_P	100	35.5	400	96	1000	[9]
4	Fe_2_O_3_//Ni_5_P_4_	88.3	29.8	400	86	1000	[9]
5	Ni_2_P NS/NF//AC	96	26	337	91.3	5000	[7]
6	AC//Ni_2_P	239 C g^−1^	53	399	85.3	3000	[6]
7	Co_2_P//GN	76.8	24	3000	97	6000	[4]
8	NCoNiP@NCoNiP//HPC	133	44	150	77	7000	[3]
9	NCoNiP//HPC	93	29.6	11,041	93.5	10,000	[2]
10	Ni_2*p*_//rP@rGO	117.18	41.66	1200	88	5000	This work

After analyzing the possible basic electrochemistry of the assembled asymmetric supercapacitor device, the next step involved the determination of its cyclic stability, i.e., its energy and power densities, which are important factors that are considered when determining the possible applications of such hybrid devices. The cyclic stability of the assembled device under 5000 consecutive charge/discharge cycles was analyzed, and the results presented in Fig. 10d showed that initially, the capacitance values gradually increased during the charge/discharge measurements, then slowly decreased, and were maintained up to 88% after the 5000 cyclic runs. This behavior is a common activation process of electrodes.

The behavior of the assembled electrodes with respect to charge transfer resistance and the ion diffusion properties of the Ni_2_P and rP@rGO device before and after the cyclic stability tests was also investigated using EIS. The results presented in Fig. 10e showed that the Nyquist plots comprised an arc in the high-frequency region, and an almost straight line in the low-frequency region. It is obvious that the impedance plot increases sharply in the low-frequency region and tends to become a straight line, suggesting the actual capacitive behavior of the electrode. However, the high-frequency region generally represents the equivalent series resistance originating from the combination of the intrinsic resistance of the active material, solution resistance, and contact resistance at the interface. The semicircular part shows the charge transfer resistance at the electrode/electrolyte interface. To fit the impedance curve with the capacitance resistance (CPE), equivalent resistance (*R*_s_), charge transfer resistance (*R*_ct_), and ionic diffusion (*W*), the equivalent circuit of the device was used. The fitting results showed that the equivalent resistance of the Ni_2_P and rP@rGO electrodes before and after the 5000 cyclic tests was 1.2 and 0.9, respectively. These results did not significantly change throughout the 5000 cycles, suggesting good electrical conductivity and an ion diffusion, an observation that is also supported by charge/discharge cycles, and are suggestive of a good capacitance retention and a high stability for the device. However, the charge transfer resistance of the electrodes before and after the 5000 cyclic tests was 18.5 and 24.8, respectively, clearly demonstrating that after 5000 cycles, the *R*_ct_ value increased compared to its value before cyclic stability test. These results demonstrate that before the cyclic test, the assembled device showed charge transfer rates that were more favorable than those recorded after the 5000 cyclic stability test. Additionally, the steeper straight line obtained before the cyclic test in the low-frequency region represent the smaller ion diffusion distance between the electrode surface and electrolyte.

**Fig. 11:**
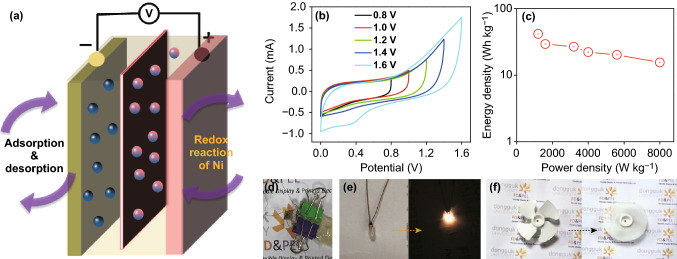
**a** Schematic diagram of the assembled asymmetric solid-state device. **b** CV voltammograms of the assembled device at various potentials. **c** Ragone plot of the solid-state device. **d** Picture of the assembled device. **e**, **f** Illumination of the LEDs and fan powered by the assembled device

The charge/discharge mechanism that occurred inside the assembled electrode can be explained as follows (Fig. 11a): during the charging process, the negative ions (OH^−^) and positive ions (K^+^) of the gel electrolyte migrate to the oppositely charged Ni_2_P and rP@rGO electrodes, respectively. This facilitates the oxidation of nickel (II) to nickel (III), as well as charge separation at the anode. Similarly, during the discharge process, the oxidized ion is reduced at the cathode, and the charges that were separated at the anode get back into the gel electrolyte. Ion migration from the anode to the cathode is responsible for triggering or glowing the LEDs. As already mentioned regarding the porous behavior of the electrode, the pores in the electrode could accommodate a numbers of ions; thus, facilitating oxidation and charge separation, which help enhance the charge storing performance of the device.

Figure 11b, which presents the CV of the asymmetric device at different voltage windows, shows the rectangular behavior of the device, which was maintained even at higher voltage values. However, a redox peak observed at a higher voltage might be due to the oxidation of OH^−^ ions, given that the gel electrolyte contains KOH.

The estimated high energy and power densities of the asymmetric supercapacitor were 41.66 and 1200 Wh kg^−1^, respectively, and an energy density of 15.55 Wh kg^−1^ was maintained at a high power density of 8000 W kg^−1^ (Fig. 11c), demonstrating the excellent high power performance of the device for targeted applications. Additionally, this energy density value was also significantly higher than those of other asymmetric devices that have been previously reported (Table 1).

To test the asymmetric supercapacitor as a commercial device, commercially available LEDs and fans were powered using the assembled device (Fig. 11d–f). The assembled device was first charged using a 9 V battery, and quickly connected to the LED, and powered for 40 s (Figs. 11d–f and S9). Similarly, the 1 W fan was also connected to the device, and powered for 30 s (Fig. S10). The results clearly showed that the charged solid-state asymmetric device could power the LED and the 1 W fan, implying that it could be used practically. This proof-of-principle demonstration of the device characteristics highlights the possible applications in the device in the field of energy storage electrode materials.

To demonstrate the importance of this study with respect to its context, the performance of the assembled device was compared with those of previously manufactured asymmetric solid-state devices. This comparison revealed that the performance of our device was superior to those of previously reported Ni_2*p*_*-* and carbon-based devices (Table 1). Another interesting observation that was made is that only four to five studies on asymmetric Ni_2_P and graphene electrode-based devices have been published. This also highlights the importance of this study in terms of the application.

Generally, the ease of the positive electrode synthesis method, as well as its outstanding electrochemical performance, can be explained by taking the following points into consideration: (1) The sufficient conductivity of Ni_2_P is associated with the presence of nickel. (2) The electronic state of nickel, which is at the origin of its pseudo-capacitance make its electrochemical applications diverse, especially in supercapacitors. On the other hand, the negative electrode, which had many visible pores that were further confirmed using analytic methods, including SEM, TEM, and HRTEM, was prepared using a hydrothermal method, and its performance was further tested in a three-electrode assembly cell. Its excellent supercapacitance could be attributed to: (1) The outstanding electronic conductivity of graphene that is incorporated into noble metals and metal oxides with double-layer material. (2) The core structure of the rP@rGO, which provides enough space for electrolyte ion adsorption during the electrochemical process, and enhances the overall capacitance. (3) The interfacial interaction between the rP and conductive rGO, which provides an easy pathway during electronic transportation, increasing the overall conductivity of the electrode. (4) The presence of rP, which prevents electroactive material agglomeration.

After combining these individual positive and negative electrode characteristics, the solid-state device showed a higher capacitance and power density that might be attributed to the porous structure of the negative electrode, which improves storage capability, and eases ion accessibility. The pure phase of the positive electrode, which has a higher specific surface area, and a higher faradic behavior, resulted in a higher capacitance and cyclic stability. The size of the 3D rP@rGO electrode, which has a porous structure, in the asymmetric solid-state device was comparable with that of the electrolyte-soaked cellulose filter paper. This helped to provide a shortcut path for ion transport between neighboring graphene layers. These characteristic permitted the assembled device to power LEDs and fans as already mentioned above.

## Conclusions

This study focused on the design of positive and negative electrodes for use in an asymmetric device using a simple and one-step synthesis method. Based on the existing literature, this study is the first to use a rP@rGO-based material as the negative electrode in an asymmetric supercapacitive solid-state device. The prepared electrode materials were characterized in detail using various microscopic and spectroscopic techniques. Additionally, an investigation of the electrochemical supercapacitive performance of the individual electrodes in a three-electrode assembly cell showed that the prepared electrodes exhibited high capacitance values, and based on the existing literature, these values are the highest ever reported from studies involving Ni_2_P-based electrodes. After the investigation of their individual electrochemical performances, the solid-state supercapacitive device was obtained by sandwiching a gel electrolyte-soaked cellulose filter paper with these electrodes. The assembled device exhibited an excellent electrochemical performance with a large potential window up to 1.6 V, and displayed a significantly high power density and energy density, as well as an excellent cyclic stability. Additionally, it could power commercially available LEDs and fans, highlighting the possibility of its application in various energy storage fields. This enhanced performance of the individual electrodes and the assembled device can be attributed to the presence of nickel and phosphorus, which play a role in the enhancement of the overall conductivity. The porous nature rP@rGO and its 3D structure provide sufficient space and reduce the path length for ion transportation in the electrolyte during the electrochemical process. The addition of rP to rGO also significantly increased capacitance performance, owing to the high theoretical capacitance of the rP. Conclusively, the results of this study indicate that rP@rGO can be a promising and excellent electrode material for application in the manufacture of different energy storage devices.

## Electronic supplementary material

Below is the link to the electronic supplementary material.

Supplementary material 1 (DOCX 11736 kb)
