# Supplementary material for: Newly Design Porous/Sponge Red Phosphorus@Graphene and Highly Conductive Ni2P Electrode for Asymmetric Solid State Supercapacitive Device With Excellent Performance
Source: Nanomicro Lett. 2020 Jan 17;12:25. doi: 10.1007/s40820-019-0360-3 (PMC7770815; doi:10.1007/s40820-019-0360-3)
Supplement: Supplementary file 1 — Supplementary material 1 (DOCX 11736 kb) [file 40820_2019_360_MOESM1_ESM.docx]

**Electronic Supplementary Information**

**Newly Design Porous/Sponge Red Phosphorus@Graphene and Highly Conductive Ni_2_P Electrode for Asymmetric Solid State Supercapacitive Device With Excellent Performance**

| S.  No. | Electrode material | Electrolyte | Current density  ( Ag^-1^) | Specific capacitance  (Fg^−1^) | Retention % | No of  cycles | Ref. |
| --- | --- | --- | --- | --- | --- | --- | --- |
| 1 | Ni_2_P | 2.0M LiOH | 1 | 418 |  |  | 1 |
| 2 | Ni-coated Ni_2_P | 2.0M LiOH | 1 | 581 | 92.3 | 3,000 | 1 |
| 3 | Ni_2_P nano belt | 0.5M H_2_SO_4_ | 0.625 | 1074 | 86.7 | 3000 | 2 |
| 4 | Ni_2_P/Ni_12_P_5_ | 2M KOH | 1 | 1325.7 | 81 | 20,000 | 3 |
| 5 | Ni-P | 2.0 M KOH | 1 | 1338.7 | 71.4 | 1,000 | 4 |
| 6 | Ni_2_P | 2.0 M KOH | 1 | 843.3 | 100 | 1000 | 5 |
| 7 | Au/Ni_12_P_5_ | 2.0 M KOH | 0.2 | 806.1 | 91.0 | 1,000 | 6 |
| 8 | Ni_2_P/rGO | 2.0 M KOH | 1 | 2354 | 100 | 2,500 | 7 |
| 9 | Ni_2_P NS/NF | 6.0 M KOH | 2.5 | 3496 | 61.0 | 5,000 | 8 |
| 10 | Ni_2_P | 2.0 M KOH | 1 | 600 | 82.5 | 1,000 | 9 |
| 11 | Ni2P@5%GR | 3 M KOH | 1 | 672.4 | 30 | 2000 | 10 |
| 12 | Ni_2_P nano particle | 3M KOH | 1 | 668.7 | -- | -- | 11 |
| 13 | Ni_2_P | 2M KOH | 1 | 1526.6 | 88 | 2,500 | Present work |
| 14 | Ni_2_p | 2M KOH | 1 | 980 | -- | -- | Present work |

Nazish Parveen,^1^ Muhammed Hilal,^2^ and Jeong In Han^*2^

^1^Department of Chemistry, College of Science, King Faisal University, Al-Ahsa 31982,

Saudi Arabia

^2^Flexible display and printed electronic lab, Department of Chemical and Biochemical

Engineering, Dongguk University-Seoul, 04620 Seoul, Republic of Korea

**Table. S1** Performance comparison of the nickel phosphide-based electrode materials in three-electrode configuration with previously published results.

**Picture of the rP@rGO foam**


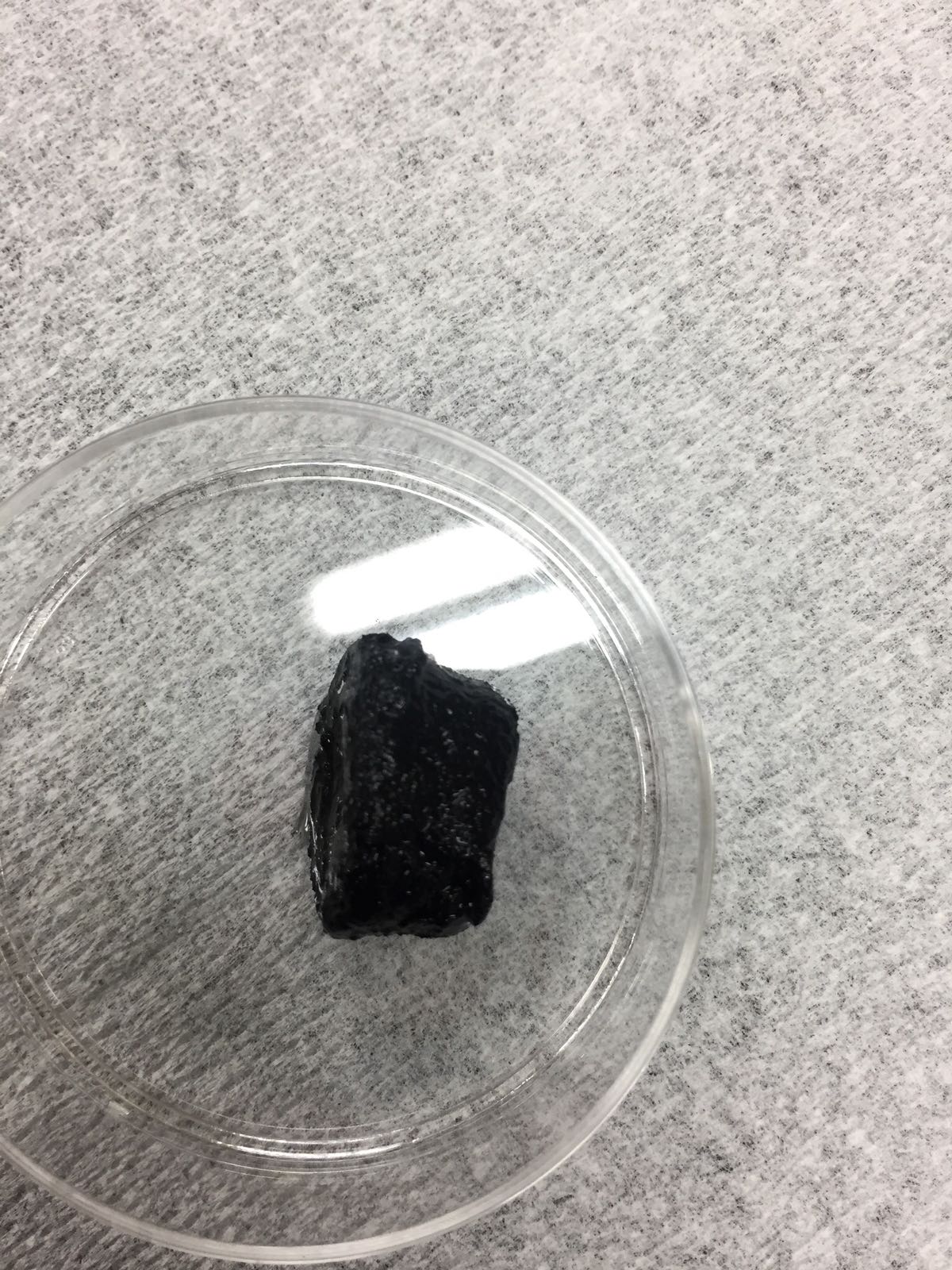


**Figure. S1** Picture of red phosphorus@graphene foam.

**Assembled asymmetric supercapacitive device**


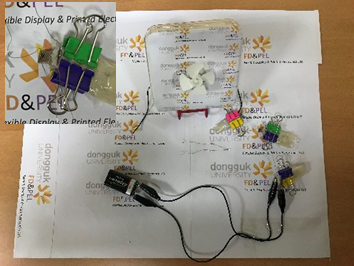


**Figure. S2** Picture of assembled asymmetric supercapacitive device**.**

**SEM, TEM, and HRTEM images of the B-rGO**

**Figure. S3** (**a-b**) SEM images (**c-d**) TEM image and (**e**) HRTEM images of the B-rGO.

**Survey spectra of the rP@rGO**

**Figure. S4** XPS surway spectra of the rP@rGO.

**O 1s spectra of the rP@rGO**

**Figure. S5** O 1s spectra of the rP@rGO.

**SEM and mapping images of the Ni_2_P-1**

**Figure S6.** (**a** and **b**) SEM images, and (**c-f**) corresponding elemental mapping of the Ni_2_P-1.

**XPS spectra of Ni_2_P-2**

**Figure S7.** High magnification XPS spectra of Ni_2_P (a) Ni and (b) P.

To estimate the specific capacitance of the as prepared positive and negative electrode inside the half-cell assembly the following equation were used.^3,5^

$C=\frac{Idt}{mdV}$ (S1)

Where C is the specific capacitance (F/g), *I* is the applied current, t is the discharge time, m represent the mass of the active materials over the surface of the current collector, and *d*V is the applied potential window.

To estimate the specific capacitance of the assembled asymmetric supercapacitor gadget the following equation are used.^3,5^

$C=\frac{Idt}{mdV}$ (S2)

Where C is the specific capacitance (F/g), *I* is the applied current, t is the discharging time, m is active mass loading over the surface of the current collector, and *d*V is the applied potential window.

Whereas the power density and energy density were estimated from the following equation:^5^

$E=\frac{1}{2}CV^{2}$ (S3)

$P=\frac{E}{t}$ (S4)

Where C is the specific capacitance, V is the applied potential window and t is the discharging time of the device.

**The CV profile of all the Ni_2_P electrodes**

**Figure S8.** The CV (**a**) and CD (**b**) profile of Ni_2_P-1electrode.

**Picture of the assembled device illumination of the LED light**

**Figure S9. (a-d)** Picture of the assembled device illumination of the LED light.

**Picture of the assembled device and fan powered by the assembled device**

**Figure S10.** Picture of the assembled device and fan powered by the assembled device.

**References**

1. Y. Lu, J. K. Liu, X. Y. Liu, S. Huang, T. Q. Wang, X.l. Wang, C. D. Gu, J. P Tu and S. X. Mao, Facile synthesis of Ni-coated Ni_2_P for supercapacitor applications, Cryst Eng Comm, 2013, 15, 7071.
2. Y. Jin a, C. Zhao, L. Wang, Q. Jiang, C. Ji, X. He, Preparation of mesoporous Ni_2_P nanobelts with high performance for electrocatalytic hydrogen evolution and supercapacitor, , international journal of hydrogen energy 2018,43, 3697-3704.
3. S. Xie, J. Go, Facile synthesis of Ni_2_P/Ni_12_P_5_ composite as long-life electrode material for hybrid supercapacitor, Journal of Alloys and Compounds, 2017, 713, 10-17.
4. D. Wang, L. B. Kong, M. Liu, W. B. Zhang, Y. C. Luo, L. Kang, Amorphous Ni–P materials for high performance pseudocapacitors, Journal of Power Sources, 2015, 274, 1107-1113.
5. D. Wang, L. B. Kong, M. C. Liu, Y. C. Luo, L. Kang, An Approach to Preparing Ni–P with Different Phases for Use as Supercapacitor Electrode Materials, Chem. Eur.J.2015, 21,17897 –17903
6. S. Duan and R. Wang, Au/Ni_12_P_5_ core/shell nanocrystals from bimetallic heterostructures: in situ synthesis, evolution and supercapacitor properties, NPG Asia Materials, 2014, 6, 122.
7. C. An, Y. Wang, Y. Wang, G. Liu, L. Li, F. Qiu, Y. Xu, L. Jiao and H. Yuana, Facile synthesis and superior supercapacitor performances of Ni_2_P/rGO nanoparticles, RSC Adv., 2013, 3, 4628-4633
8. K. Zhou, W. Zhou, L. Yang, J. Lu, S. Cheng, W. Mai, Z. Tang, L. Li, S. Chen, Ultrahigh‐Performance Pseudocapacitor Electrodes Based on Transition Metal Phosphide Nanosheets Array via Phosphorization: A General and Effective Approach, Advanced Functional Materials, 2015, [25,](https://onlinelibrary.wiley.com/toc/16163028/2015/25/48) 7530-7538.
9. M. C. Liu, Y. M. Hu, W. Y. An, Y. X. Hu, L. Y. Niu, L. B. Kong, L. Kang, Construction of high electrical conductive nickel phosphide alloys with controllable crystalline phase for advanced energy storage, Electrochimica Acta, 2017, 232, 387–395
10. W. Du, S. Wei, K. Zhou, J. Guo, H. Pang, X. Qian, One-step synthesis and graphene-modification to achieve nickel phosphide nanoparticles with electrochemical properties suitable for supercapacitors, Materials Research Bulletin, 2015, 61, 333–339.
11. W. Du, R. Kang, P. Geng, X. D. Li, Q. Tian, H. Pang, New asymmetric and symmetric supercapacitor cells based on nickel phosphide nanoparticles, Materials Chemistry and Physics, 2015, 165, 207-214.
